# Physiological and Psychological Effects on High School Students of Viewing Real and Artificial Pansies

**DOI:** 10.3390/ijerph120302521

**Published:** 2015-02-25

**Authors:** Miho Igarashi, Mariko Aga, Harumi Ikei, Takafumi Namekawa, Yoshifumi Miyazaki

**Affiliations:** 1 Center for Environment, Health and Field Sciences, Chiba University, 6-2-1 Kashiwa-no-ha, Kashiwa, Chiba 277-0882, Japan; E-Mails: miho.murachi@gmail.com (M.I.); marikoaga@gmail.com (M.A.); ikei.harumi@gmail.com (H.I.); 2 Chiba Prefectural Kashiwanoha Senior High School, 6-1 Kashiwa-no-ha, Kashiwa, Chiba 277-0882, Japan; E-Mail: tname2@gmail.com

**Keywords:** flowers, artificial flowers, horticultural therapy, high school students, stress reduction, heart rate variability

## Abstract

The relaxation effects of gardening have attracted attention; however, very few studies have researched its physiological effects on humans. This study aimed to clarify the physiological and psychological effects on high school students of viewing real and artificial pansies. Forty high school students (male: 19, female: 21) at Chiba Prefectural Kashiwanoha Senior High School, Japan, participated in this experiment. The subjects were presented with a visual stimulation of fresh yellow pansies (*Viola x* *wittrockiana* “Nature Clear Lemon”) in a planter for 3 min. Artificial yellow pansies in a planter were used as the control. Heart rate variability was used as a physiological measurement and the modified semantic differential method was used for subjective evaluation. Compared with artificial pansies, visual stimulation with real flowers resulted in a significant decrease in the ratio of low- to high-frequency heart rate variability component, which reflects sympathetic nerve activity. In contrast, high frequency, which reflects parasympathetic nerve activity, showed no significant difference. With regard to the psychological indices, viewing real flowers resulted in “comfortable”, “relaxed”, and “natural” feelings. The findings indicate that visual stimulation with real pansies induced physiological and psychological relaxation effects in high school students.

## 1. Introduction

In the increasingly urbanized and technological modern society, mitigation of stress is a great concern and much attention has been paid to the relaxation effects of the natural environment. Humans emerged millions of years ago [1,2] and evolved while adapting to natural environments; therefore, human stress states are believed to be mitigated by contact with natural environments [3,4,5]. Miyazaki *et al.* (2011) mentioned that humans who live in modern society have experienced more than 99.9% of the evolutionary process in natural environments [4]. Our bodies, which are adjusted to natural environments, cannot respond well to the suddenly developed artificial society; thus, we always feel stressed. For example, an epidemiological investigation that focused on a green tract of land showed that the area had low mortality of residents [6]. Another study pointed out the importance of a green tract of land in urban residential environments [7].

In cities, gardening enables easy contact with natural environments and has received considerable attention [8]. School and community gardens are used for improving stress states and for rehabilitation in hospitals or welfare facilities [9]. However, studies on the effects of gardening on humans have been limited to psychological indices such as questionnaires; very rarely have studies reported on the physiological evaluation of the effects of gardening. Some psychological evaluations have reported the use of gardening in rehabilitation, e.g., in older people ([10,11,12,13,14,15,16], patients with clinical depression [17,18], those with substance abuse [19], patients in an inpatient cardiopulmonary rehabilitation program [20], and patients with brain damage [21]. Some reports have also indicated the health benefits of community gardens [22,23,24] and schoolyards [25]. However, few studies have measured the physiological effects of gardening. Van den Berg and Custers (2011) reported that salivary cortisol levels are decreased by gardening activity [26]. However, they used indoor reading as a control, which may compromise broad interpretation of the results. A study of fresh chrysanthemum transplantation [27] revealed no significant differences among subjects; however, the authors indicated significant differences according to personality classifications, which we believe was a limitation.

We have studied the effects of nature-derived stimulation, such as that of a forest [28,29,30,31,32,33,34,35,36], parks [37,38], a rooftop garden [39], flowers [40,41,42], foliage plants [43,44], and olfactory stimulation [45,46,47,48,49]. We observed that these natural environments and plants have physiological relaxation effects and can reduce stress states. In this study, using heart rate variability (HRV) by fingertip acceleration pulse wave, we elucidated the physiological effects of visual stimulation with flowers in a gardening activity.

We focused on the stress state of minors (high school students) and studied the physiological effects of viewing flowers in a gardening activity. Some reports have stated that the numbers of high school students with stress and uneasiness have increased in Japan [50], the United States [51], and England [52].

Therefore, to clarify the physiological effects of visual stimulation with pansies, which are the most wholesale variety of flower beds, we recruited Japanese high school students and recorded their HRV using fingertip acceleration pulse wave.

## 2. Experimental Section

We conducted this study in Chiba Prefectural Kashiwanoha Senior High School on October 30 and November 1, 2, and 5, 2012. The average temperature, humidity, and intensity of illumination in a classroom were 22.5 °C ± 1.1 °C, 50.9% ± 5.3%, and 1030 lx, respectively. The participants were 19 healthy high school boys (mean age ± standard deviation: 16.2 ± 0.7 years) and 21 healthy high school girls (mean age ± standard deviation: 16.6 ± 0.9 years).

All subjects agreed to the study protocol and provided written informed consent. The study was conducted according to the regulations of the Ethics Committee of the Center for Environment, Health, and Field Sciences, Chiba University, Japan.

**Figure 1:**
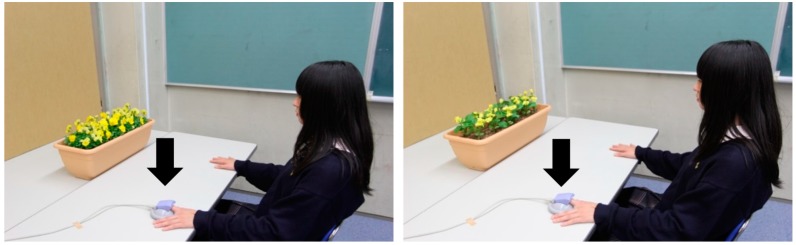
Visual stimulation with real flowers (left) and artificial flowers (right). The black arrow indicates the accelerated plethysmograph (ARTETT; U-medica, Inc., Co.).

Figure 1 shows the experimental design. After listening to the details of the experiment in a classroom different from the experimental room, the subjects practiced subjective evaluation. Then, they moved into the experimental room and formed pairs. In a seated position, the subjects rested for 1 min in front of a cardboard box covering pansies in a planter. The subjects then received visual stimulation with either real pansies (hereafter real flowers) (Figure 1, left) or artificial pansies (hereafter artificial flowers) (Figure 1, right) for 3 min. We measured fingertip accelerated plethysmography and pulse rate continually during the experiment. After visual stimulation, subjective evaluation was performed. The student pairs took turns in the visual stimulation, providing counterbalance between subjects.

For visual stimulation, we used fresh yellow pansies (*Viola x wittrockiana*, “Nature Clear Lemon”). The pansies’ scent was evaluated as “weak” in terms of the sense of strength and “slightly good” in the sensuality evaluation by the modified semantic differential (SD) method. The artificial flowers were yellow pansies made of polyester. Twelve real and 12 artificial flowering plants were set with gardening soil in a polypropylene planter (55 cm long, 21.5 cm wide, 18 cm high).

As a physiological measurement, HRV was calculated by spectral analysis of the coefficient of variation of the a-a interval on an accelerated plethysmograph (ARTETT, U-Medica Inc., Osaka, Japan) [53], and the pulse rates were converted by a 60/a-a interval. The sampling frequency was 1000 Hz. The power levels of the high-frequency (HF) (0.15–0.40 Hz) and low-frequency (LF) (0.04–0.15 Hz) HRV components were calculated by the maximum entropy method (MemCalc/Win; GMS) [54]. The HF power was considered to reflect parasympathetic nerve activity, and the LF/HF power ratio was determined to reflect sympathetic nerve activity [55,56]. The subjects placed their left forefingers on the sensor of an accelerated plethysmograph and measurements were performed continuously for 3 min during visual stimulation. Concerning HRV and pulse rate measurement by plethysmography, we calculated time-dependent changes every minute with a mean of 3 min.

Evaluation by the modified SD method [57] was performed using 3 pairs of adjectives on 13 scales, including “comfortable-uncomfortable”, “relaxed-awakening”, and “natural-artificial”.

Statistical analysis was performed using SPSS 20.0 (IBM Corp., Armonk, NY, USA). A paired *t* test compared the differences in the mean physiological data, and a value of *p* < 0.05 was considered to indicate statistical significance. The Wilcoxon signed-rank test was used to analyze differences in the psychological indices, and values of *p* < 0.01 were considered to indicate statistical significance. A one-sided test was used in this study because of the hypothesis that humans would be relaxed by viewing real flowers.

## 3. Results

**Figure 2:**
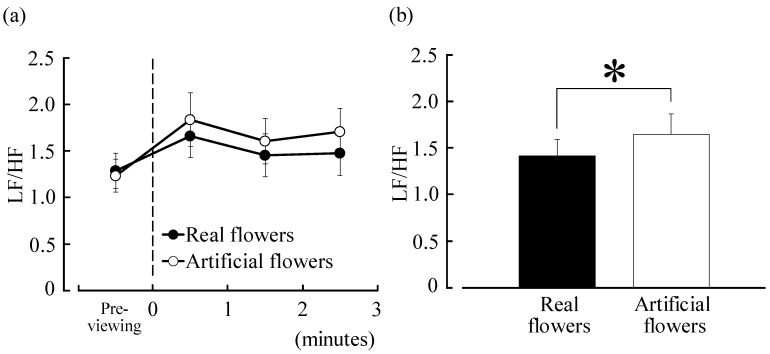
(**a**) Change in the low-frequency (LF)/high-frequency (HF) values of heart rate variability (HRV) between real and artificial flowers; (**b**) Comparison of LF/HF values of HRV between real and artificial flowers. *n* = 40, mean ± SE. * *p* < 0.05, determined by paired *t*-test (one-sided).

Time-dependent LF/HF, which reflects sympathetic nerve activity, changed every minute during visual stimulation with real flowers or control, as shown in Figure 2a. Visual stimulation with real flowers showed lower values than that with artificial flowers (0–1 min, real flowers: 1.66 ± 0.23, artificial flowers: 1.84 ± 0.29; 1–2 min, real flowers: 1.45 ± 0.23, artificial flowers: 1.61 ± 0.24; and 2–3 min, real flowers: 1.48 ± 0.24, artificial flowers: 1.71 ± 0.25). Real flowers showed lower movement than artificial flowers: at 0–1 min, 9.6% lower; 1–2 min, 9.6% lower; 2–3 min, 13.4% lower. The mean LF/HF value for 3 min is shown in Figure 2b. The value for real flowers was 1.42 ± 0.17, whereas that for artificial flowers was 1.64 ± 0.22; hence, real flowers showed a significant decrease of 13.8% (*p* < 0.05). The pre-value for real flowers and artificial flowers was 1.29 ± 0.19 and 1.23 ± 0.18, respectively, indicating no significant difference. In contrast, HF (which is considered to reflect parasympathetic nerve activity) and pulse rate did not show a significant difference.

**Figure 3:**
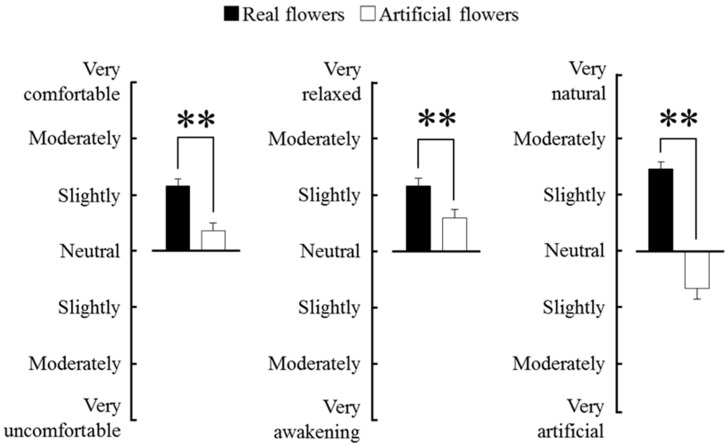
Comparison of the subjective scoring for comfortable, relaxed, and natural feelings between real and artificial flowers. *n* = 40, mean ± SE. ** *p* < 0.01, determined by Wilcoxon signed-rank test (one-sided).

A modified SD method was used for subjective evaluation of “comfortable”, “relaxed”, and “natural” feelings (Figure 3). Subjective evaluation of “comfortable” indicated that subjects felt “slightly comfortable” with real flowers. The difference between real flowers and artificial flowers was statistically significant (*p* < 0.01), with the subjects feeling more comfortable with real flowers than with artificial flowers. In terms of “relaxed” feeling, the subjects felt “slightly relaxed” by real flowers. The difference between real flowers and artificial flowers was statistically significant (*p* < 0.01), with the subjects feeling more relaxed with real flowers than with artificial flowers. In terms of “natural” feeling, the subjects felt “slightly natural” to “moderately natural” with real flowers. In this respect, the difference between real flowers and artificial flowers was statistically significant (*p* < 0.01), with the subjects feeling “more natural” with real flowers than with artificial flowers.

## 4. Discussion

We evaluated the physiological effects of visual stimulation with real and artificial pansies according to autonomic nerve activity. Compared with artificial flowers, real flowers significantly decreased sympathetic nerve activity. This result resembles that reported by a previous study on chrysanthemum transplantation for male university students with the Type A behavior pattern [27]. In contrast, some previous studies have reported increased parasympathetic nerve activity in addition to decreased sympathetic nerve activity, e.g., when viewing a forest environment in a sitting position [29,31,33,34] and following visual stimulation of high school students with fresh roses [41]. In addition, in some reports, only parasympathetic nerve activity increased, e.g., when viewing a forest environment in a sitting position [32] and following visual stimulation of office workers [40] or medical workers [42] with fresh roses. The cause of these differences remains unclear, and we intend to determine the cause(s) in future studies. With regard to the pulse rate, no significant difference was observed between the effect of real and artificial flowers. This result corresponds with that reported by previous studies, e.g., the chrysanthemum transplantation experiment [26] and the visual stimulation of high school students [41] or office workers [40] with fresh roses, which indicated no significant difference in pulse rate but a significant difference in HRV.

With regard to the psychological effects evaluated by the modified SD method, visual stimulation with real flowers resulted in “comfortable”, “relaxed,” and “natural” feelings. These results correspond with those of previous studies. In addition, few previous studies used the modified SD method on gardening activity, but chrysanthemum transplantation felt “comfortable”, “soothed”, and “natural” [41].

Considerable attention is being paid to the effect of forest environment on humans. In addition to psychological indices, comprehensive evaluation using physiological indices has been performed for forests, indicating that the forest environment has relaxation effects [28,29,32,33,34,35,36]. In contrast, with respect to gardening activity, many studies have reported the associated psychological effects [10,11,12,13,14,15,16,17,18,19,20,21,22,23,24,25]. However, knowledge regarding the physiological effects is insufficient. To elucidate the effects of gardening activity, comprehensive evaluation using both physiological and psychological indices is required. Hence, this study elucidated the relaxation effects of real flowers, used in a gardening activity, in terms of both physiological and psychological indices. The real pansies’ odor was evaluated as “weak” in this study. However, a few previous studies have reported on the influence of odor on choice and preference even when the odor could not be detected at the olfactory level [58,59,60]. The potential influence of the weak odor of pansies on the physiological response or subjective evaluation remains to be clarified in a further study. In addition, the result may have been biased because of the knowledge of fresh flowers being “natural” and artificial flower being “unreal”. In future studies, we aim to evaluate some aspects of nature connectedness and determine how these pre-determined ideas may influence the results.

In conclusion, compared with artificial pansies, visual stimulation with fresh pansies decreased sympathetic nerve activity and the subjects felt comfortable, relaxed, and natural. Visual stimulation with fresh pansies induced physiological and psychological relaxation effects on high school students.

This study has some limitations. First, high school students were the subjects in this study; studies with subjects from various ages and environments are required. Second, we used autonomic nerve activity as a physiological measurement. Other indices such as brain activity will be required from a perspective of systemic collaboration. Third, we focused on visual stimulation. However, we believe that the physiological effects of actually touching plants, such as transplantation, are important study themes.

We believe that this study provides important findings about the relationship between humans and natural environments.

## 5. Conclusions

Compared with artificial pansies, visual stimulation with fresh pansies decreased sympathetic nerve activity and the subjects felt comfortable, relaxed, and natural. The findings indicate that visual stimulation with fresh pansies induced physiological and psychological relaxation effects on high school students.
